# Association between glycemic variability and the risk of acute kidney injury in patients with traumatic brain injury: a retrospective cohort study with independent cohort analysis

**DOI:** 10.3389/fneur.2026.1797958

**Published:** 2026-04-13

**Authors:** Yajing Feng, Lizhen Liu, Yinlong Ren, Zhuoji Li, Yutong Liu, Longyu Jiang, Yuchun Liu, Junbing He, Yiming Shao

**Affiliations:** 1 The Intensive Care Unit, Affiliated Hospital of Guangdong Medical University, Zhanjiang, Guangdong, China; 2 The Intensive Care Unit, The First Dongguan Affiliated Hospital, Guangdong Medical University, Dongguan, Guangdong, China; 3 The Key Laboratory of Sepsis Translational Medicine, Guangdong Medical University, Zhanjiang, Guangdong, China; 4 Department of Intensive Care Unit, The First Affiliated Hospital of Jinan University, Guangzhou, China; 5 The Key Laboratory of Organ Dysfunction and Protection Translational Medicine, Jieyang Medical Research Center, Jieyang People’s Hospital, Jieyang, Guangdong, China

**Keywords:** acute kidney injury, critical care, glycemic variability, independent cohort replication, MIMIC-IV database, traumatic brain injury

## Abstract

**Background:**

Acute kidney injury (AKI) is a common complication among critically ill patients with traumatic brain injury (TBI) and is associated with adverse clinical outcomes. Glycemic variability (GV), reflecting short-term fluctuations in blood glucose, may contribute to organ dysfunction; however, its relationship with AKI in ICU patients with TBI remains incompletely characterized.

**Methods:**

We conducted a retrospective cohort study using the MIMIC-IV database, including 2,151 adult ICU patients with TBI, and performed replication in an independent cohort of 265 patients to evaluate reproducibility and incremental prognostic value. GV was quantified as the coefficient of variation (CV) of glucose measurements obtained during ICU stay until the occurrence of AKI or ICU discharge. Multivariable logistic regression models were used to examine the association between GV and AKI, with stepwise adjustment for potential confounders. Predictive performance was assessed using receiver operating characteristic (ROC) analysis, while dose–response relationships were explored with restricted cubic spline models. The incremental prognostic utility of adding GV to SOFA- and APACHE II-based models was evaluated using calibration plots, decision curve analysis, Integrated Discrimination Improvement (IDI), and Net Reclassification Improvement (NRI).

**Results:**

AKI occurred in 59.1% (1,271/2,151) of patients in the MIMIC-IV cohort. Higher GV was independently associated with AKI across all adjusted models (fully adjusted model: OR 1.16, 95% CI 1.02–1.34). ROC analysis yielded an area under the curve of 0.73 (95% CI 0.71–0.75) with 66% sensitivity and 69.7% specificity. Restricted cubic spline analyses suggested an approximately linear increase in AKI risk with rising GV. The association was consistent across prespecified subgroups, robust to adjustment for glucose monitoring intensity, and replicated in the independent cohort. Incorporating GV into SOFA- and APACHE II-based models led to modest but statistically significant improvements in risk stratification.

**Conclusion:**

In ICU patients with TBI, greater glycemic variability is independently associated with subsequent AKI and provides incremental prognostic information beyond established severity scores. These findings highlight the potential utility of GV as a complementary marker for risk stratification. Prospective studies using standardized GV definitions and harmonized glucose monitoring strategies are warranted to confirm these results and clarify their clinical implications.

## Introduction

TBI represents a major cause of death and long-term disability worldwide and continues to impose substantial clinical and socioeconomic burdens (1). It contributes substantially to mortality and long-term cognitive impairment in adults and is also associated with several non-neurological complications, including AKI (2). AKI is a frequent complication of TBI, with reported incidence rates ranging from 30 to 50%, and can aggravate multi-organ dysfunction and markedly prolong hospital stay (3). The interaction between the injured brain and the kidney is increasingly recognized as a bidirectional and pathophysiologically integrated process. Following TBI, sympathetic hyperactivation, hypothalamic–pituitary–adrenal axis stimulation, inflammatory cytokine release, and hemodynamic instability collectively promote renal vasoconstriction, microcirculatory dysfunction, and tubular injury (4, 5). In turn, AKI may amplify secondary brain injury through endothelial dysfunction, disruption of the blood–brain barrier, uremic toxin accumulation, and metabolic derangements (6, 7). This reciprocal “brain–kidney axis” suggests that metabolic and hemodynamic perturbations occurring in the early phase of TBI may play a critical role in precipitating renal injury. Identifying modifiable physiological signals that precede AKI could therefore provide opportunities for earlier risk stratification and targeted intervention.

Glucose dysregulation is a hallmark of the stress response in critically ill patients with TBI. Beyond sustained hyperglycemia, increasing attention has been directed toward GV, which reflects short-term fluctuations in glucose levels and may serve as a surrogate marker of acute metabolic instability. Unlike static glucose indices, GV captures dynamic oscillations driven by insulin resistance, counter-regulatory hormone surges, inflammation, and therapeutic interventions. Multiple metrics have been proposed to quantify GV, including mean amplitude of glycemic excursions (MAGE), time in range (TIR), standard deviation (SD), and CV, each representing distinct aspects of glycemic dynamics (8–11). Among these, CV has been widely applied in critical care research because it standardizes dispersion relative to mean glucose and facilitates inter-individual comparison (10, 12).

Emerging evidence suggests that glycemic variability may exert deleterious biological effects independent of mean glucose levels. Experimental and clinical studies indicate that rapid glucose fluctuations can promote oxidative stress, endothelial dysfunction, inflammatory activation, and mitochondrial injury—mechanisms that are closely implicated in the pathogenesis of AKI (13–15). In the context of TBI, stress-induced insulin resistance and catecholamine excess may further amplify glycemic oscillations, while sympathetic overactivation can concurrently induce renal vasoconstriction via α1-adrenergic pathways, thereby predisposing the kidney to ischemic injury (4, 16–18). These converging mechanisms raise the possibility that GV is not merely a metabolic epiphenomenon but a potential contributor to organ cross-talk within the brain–kidney axis.

Despite these biologically plausible links, data specifically examining the relationship between GV and AKI in patients with TBI remain limited. Most prior investigations have focused on average glucose levels or single time-point measurements, potentially overlooking the prognostic significance of dynamic metabolic fluctuations. Whether GV independently predicts AKI in critically ill TBI populations—and whether it provides incremental value beyond established severity scores—has not been systematically evaluated.

Therefore, we conducted a retrospective cohort study of ICU patients with TBI to examine the association between glycemic variability and subsequent AKI. We further assessed whether incorporating GV into conventional severity models improves risk stratification. Clarifying this relationship may refine metabolic monitoring strategies and enhance early identification of patients at risk for renal complications following TBI.

## Materials and methods

### Study design and data sources

We performed a retrospective cohort study with independent cohort replication to investigate the association between glycemic variability and acute kidney injury in critically ill patients with TBI.

The primary cohort was extracted from the Medical Information Mart for Intensive Care IV (MIMIC-IV, version 3.1), a large, publicly available critical care database comprising approximately 90,000 ICU admissions to Beth Israel Deaconess Medical Center (Boston, MA, United States) between 2008 and 2022 (19). Access to the database was granted after completion of the required training and certification (Record ID: 65740860). The MIMIC-IV database is de-identified and approved for research use by the Institutional Review Boards of the Massachusetts Institute of Technology and Beth Israel Deaconess Medical Center, with a waiver of informed consent.

To examine the reproducibility of our findings, we assembled an independent cohort from the electronic medical record system of the Affiliated Hospital of Guangdong Medical University (Guangdong, China), including consecutive ICU admissions between 1 January 2020 and 30 November 2025. The study protocol was reviewed and approved by the Ethics Committee of the Affiliated Hospital of Guangdong Medical University (PJKT2025-291), which waived the requirement for additional informed consent due to the retrospective use of anonymized data. All procedures were conducted in accordance with the Declaration of Helsinki.

### Study population

**Figure 1:**
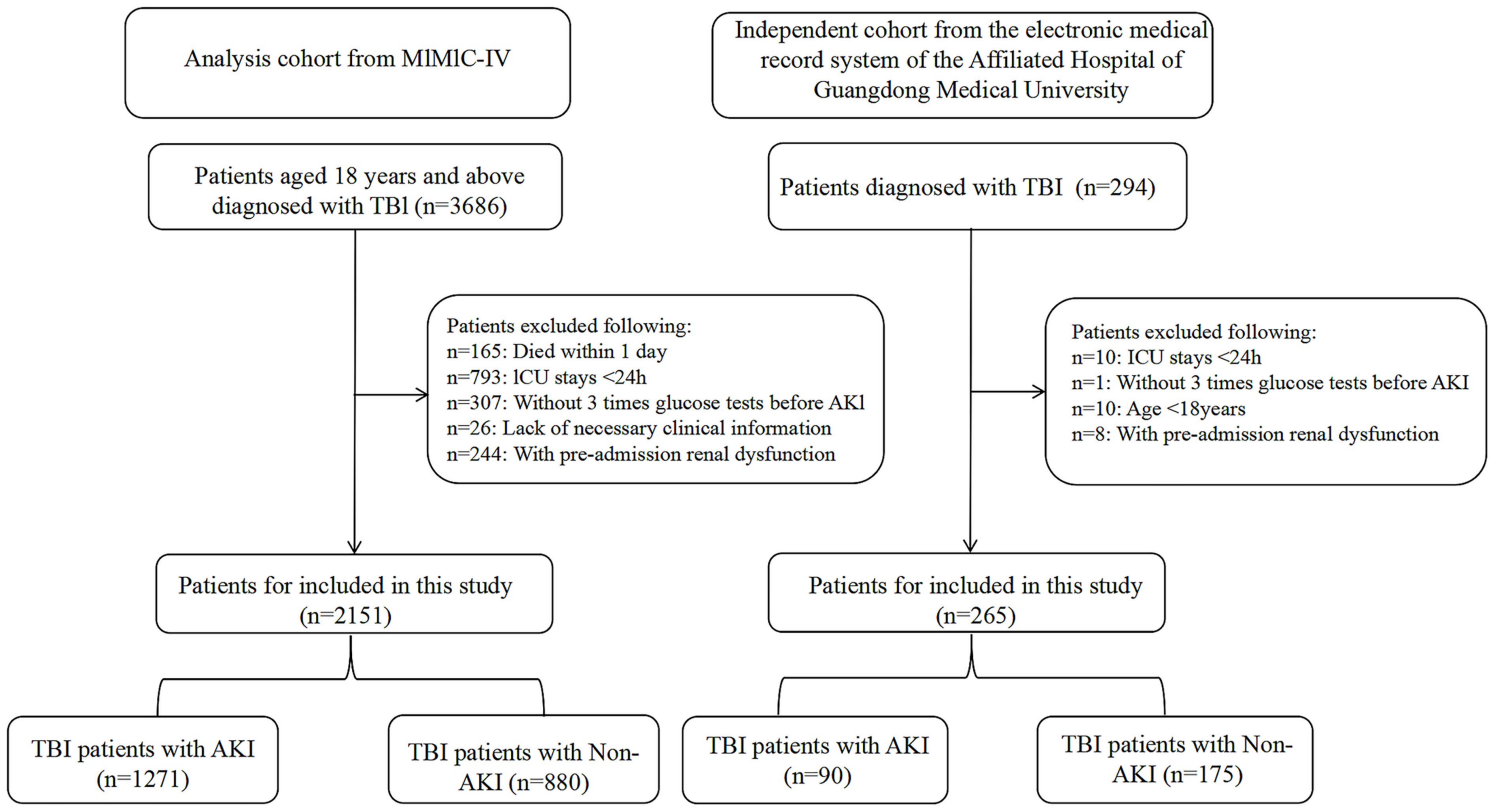
Overall flowchart of this study. MIMIC-IV, Medical Information Mart for Intensive Care IV.

Adult patients (≥18 years) with a diagnosis of TBI identified using International Classification of Diseases (ICD-9 and ICD-10) codes were eligible for inclusion. To ensure adequate assessment of glycemic variability prior to renal injury, patients were required to have: An ICU length of stay ≥24 h; and At least three blood glucose measurements recorded before the onset of AKI. Exclusion criteria were as follows: (1) Age <18 years; (2) Pre-existing chronic kidney disease at ICU admission; (3) Receipt of renal replacement therapy (RRT) or chronic dialysis prior to admission; (4) Fewer than three pre-AKI glucose measurements; (5) ICU stay <24 h; (6) Missing key ICU admission data. If multiple ICU admissions occurred, only the first eligible ICU stay was included in the analysis. After applying these criteria, 2,151 patients from the MIMIC-IV cohort and 265 patients from the independent cohort were included (Figure 1).

### Data collection and variables

Data were extracted using DecisionChain software (version 1.1.7). Baseline covariates included demographic characteristics, comorbidities, illness severity indices, laboratory variables, and therapeutic interventions recorded during the ICU stay. GV was quantified using the CV, calculated as CV = [SD of blood glucose/mean blood glucose (MBG)] × 100%, providing a normalized measure of glucose fluctuation independent of absolute glucose levels (9). Patients were required to have at least three glucose measurements within the exposure window to ensure minimally reliable estimation of dispersion-based metrics. Because variability measures are sensitive to sampling density, insufficient observations may yield unstable or artificially inflated estimates. Consistent with prior methodological work in critically ill populations (20, 21), this threshold was selected to balance statistical reliability with preservation of sample size.

### Endpoint

The primary endpoint of this study was the incidence of AKI. AKI was diagnosed according to the Kidney Disease: Improving Global Outcomes (KDIGO) criteria (22), and was defined by the presence of any of the following: (a) an increase in serum creatinine (Scr) of ≥0.3 mg/dL within 24 h; (b) an increase in Scr to ≥1.5 times the baseline value within 7 days; or (c) urine output <0.5 mL/kg/h for at least 6 h. Baseline Scr was defined as the value measured within the first 24 h after ICU admission.

### Statistical analysis

Continuous variables are reported as median [interquartile range (IQR)] and compared using the Wilcoxon rank-sum test. Categorical variables are presented as counts (percentages) and compared using the chi-square test. Variables with >20% missingness were excluded. For variables with ≤20% missingness, multiple imputation by chained equations (MICE) was applied under the missing-at-random assumption.

Univariate logistic regression was first used to identify candidate predictors of AKI. Variables meeting predefined selection criteria were then entered into multivariable logistic regression models, with GV specified as the primary exposure. To evaluate the robustness of the association, three hierarchical models were constructed: Model 1 was unadjusted; Model 2 was adjusted for covariates significant in univariate analysis, with baseline glucose and diabetes status retained regardless of statistical significance to ensure appropriate interpretation of GV-related effects; and Model 3 was fully adjusted for baseline covariates while excluding post-exposure variables potentially influenced by early renal injury (e.g., mechanical ventilation, continuous renal replacement therapy, and 28-day ICU mortality) to reduce overadjustment and reverse causation bias. Multicollinearity was assessed before multivariable modeling using variance inflation factors (VIFs), with values >10 indicating severe collinearity (23). All covariates had VIF values <10, supporting acceptable collinearity and stable model estimation (Supplementary Table 1). GV was calculated using all available blood glucose measurements recorded during the ICU stay until the occurrence of AKI or ICU discharge.

Glycemic variability was calculated using all available glucose measurements recorded during the ICU stay until the occurrence of AKI or ICU discharge, with ICU admission defined as the baseline time point. Because the number of glucose measurements may vary substantially across patients during ICU hospitalization, this approach may introduce potential time-related bias and heterogeneity in exposure assessment due to differences in monitoring intensity. Variability in monitoring frequency has been recognized as an important methodological concern in observational analyses of ICU monitoring data (24). To mitigate this potential bias, additional analytical strategies were implemented. First, the total number of glucose measurements obtained during ICU stay was included as an additional covariate in the fully adjusted multivariable regression model to account for differences in monitoring frequency across patients. In addition, glucose monitoring density was calculated as the number of glucose measurements divided by ICU length of stay (expressed as measurements per ICU day), thereby standardizing monitoring intensity relative to observation time.

Although stratification according to predefined categories of measurement counts (e.g., 5–7, 8–10, and >10 measurements) has been used in previous studies to explore monitoring-related bias, such thresholds are inherently arbitrary and may lead to imbalanced sample sizes depending on the underlying distribution of the data; this pattern was also observed in the present study (Supplementary Table 2). Moreover, heterogeneity in monitoring frequency may influence the probability of detecting abnormal values and thereby introduce spurious associations in observational analyses (24).

To reduce subjectivity and better account for variability in ICU length of stay, sensitivity analyses were conducted based on tertiles of measurement density (low, medium, and high monitoring intensity) derived from the empirical distribution of the study population. Within each measurement density tertile, multivariable logistic regression models were constructed to evaluate the association between glycemic variability and AKI while adjusting for the same prespecified covariates used in the primary analysis. In addition, formal interaction testing was performed by introducing an interaction term between glycemic variability and measurement density into the fully adjusted model to assess potential effect modification by monitoring intensity.

Discriminatory performance was evaluated using receiver operating characteristic analysis, including calculation of the area under the curve (AUC), sensitivity, and specificity, and the optimal cutoff value for GV was determined using ROC–based criteria. To assess the incremental prognostic value of GV beyond established severity scores, calibration plots (Supplementary Figure 1) and decision curve analysis were generated, and IDI together with NRI were calculated to quantify changes in risk stratification after incorporation of GV into SOFA- and APACHE II-based models (25). To further evaluate the robustness of the findings, prespecified sensitivity analyses were performed by restricting the analysis to patients with at least five glucose measurements within the exposure window, and the standard deviation of glucose values was additionally examined as an alternative metric of GV. In addition, restricted cubic spline models were used to explore potential non-linear dose–response relationships between GV and the risk of AKI. Kaplan–Meier curves were generated to evaluate 28-day ICU survival, and group differences were assessed using the log-rank test.

To explore potential heterogeneity in the association between GV and AKI, subgroup analyses were prespecified based on clinical and biological considerations, including age, gender, baseline neurological severity, and comorbid conditions relevant to glycemic regulation, vascular function, and organ vulnerability. Specifically, baseline neurological severity was stratified according to the Glasgow Coma Scale, and comorbid conditions included diabetes mellitus, sepsis, hypertension, and chronic liver disease (26–29). These subgroup variables were defined *a priori* to enhance analytical transparency and to assess potential effect modification across clinically relevant patient groups. Interaction terms were formally tested to evaluate the consistency of the association between GV and AKI across subgroups.

To further examine the reproducibility of the observed association, we conducted an independent cohort analysis using data from a separate dataset. Glycemic variability was calculated using the same predefined metrics as in the primary cohort. The optimal cut-off value was determined within the independent dataset using receiver operating characteristic analysis. Because the optimal threshold was re-estimated in the independent cohort and the original model parameters were not preserved in a fixed (“frozen”) form, this analysis does not represent strict external validation. Instead, it should be interpreted as an independent cohort analysis aimed at assessing reproducibility and the incremental prognostic utility of glycemic variability, in accordance with established methodological guidance for prediction model studies and the TRIPOD recommendations (30, 31).

All statistical analyses were performed using DecisionChain software (version 1.1.7; Hangzhou Yuantong Information Technology Co., Ltd., Hangzhou, China) (32). A two-sided *p* < 0.05 was considered statistically significant.

## Results

### Characteristics of the study population

This study analyzed data from 2,151 patients with TBI in the MIMIC-IV cohort. As shown in Table 1, patients were stratified according to the occurrence of AKI during their ICU stay: 880 patients did not develop AKI, whereas 1,271 did, corresponding to an AKI incidence of 59.1%. There were no significant differences in age or sex distribution between the AKI and non-AKI groups (*p* = 0.12 and *p* = 0.056, respectively). Compared with the non-AKI group, patients who developed AKI had higher admission heart rate, white blood cell (WBC) count, prothrombin time (PT), activated partial thromboplastin time (APTT), blood urea nitrogen (BUN), Scr, and blood glucose levels. The AKI group also had a higher prevalence of diabetes mellitus and required mechanical ventilation and CRRT more frequently. In addition, GV was significantly higher in the AKI group than in the non-AKI group (*p* < 0.001).

**Table 1:** Clinical characteristics of the study participants.

Variable names	Analysis cohort (*N* = 2,151)		*p*-value	Independent cohort (*N* = 265)		*p*-value
	Non- AKI *N* = 880	AKI *N* = 1,271		Non- AKI *N* = 175	AKI *N* = 90	
Demographic						
Age (years)	65.00 (45.00–79.00)	65.00 (48.00–80.00)	0.120	56.00 (42.00–66.00)	62.00 (49.00–73.00)	0.004
Gender			0.056			0.003
Male	535.00 (60.80%)	824.00 (64.83%)		133.00 (76.00%)	82.00 (91.11%)	
Female	345.00 (39.20%)	447.00 (35.17%)		42.00 (24.00%)	8.00 (8.89%)	
Vital signs						
Heart rate (bpm)	83.00 (72.00–95.00)	85.00 (72.00–99.00)	0.007	92.00 (77.00–105.00)	105.00 (87.00–125.00)	<0.001
Temperature (°C)	36.83 (36.56–37.17)	36.83 (36.50–37.22)	0.312	37.00 (36.60–37.30)	36.90 (36.50–37.80)	0.112
SBP (mmHg)	128.00 (115.00–143.00)	129.00 (114.00–145.00)	0.365	136.00 (116.00–158.00)	122.00 (108.00–152.00)	0.046
MBP (mmHg)	91.00 (80.83–101.33)	90.33 (80.00–101.33)	0.490	92.00 (80.00–103.00)	88.00 (75.00–99.00)	0.025
SPO_2_ (%)	99.00 (96.00–100.00)	99.00 (97.00–100.00)	0.307	100.00 (99.00–100.00)	100.00 (98.00–100.00)	0.609
Severity of illness scores						
SOFA	2.00 (2.00–4.00)	3.00 (2.00–5.00)	<0.001	6.00 (4.00–8.00)	9.00 (8.00–13.00)	<0.001
GCS	14.00 (12.00–15.00)	14.00 (12.00–15.00)	0.253	7.00 (5.00–11.00)	4.00 (3.00–7.00)	<0.001
APACHE II	12.00 (9.00–16.00)	14.00 (11.00–19.00)	<0.001	21.00 (17.00–26.00)	30.50 (25.00–36.00)	<0.001
Laboratory tests						
PLT (k/uL)	198.00 (160.00–249.50)	191.00 (144.00–245.00)	0.023	173.00 (132.00–263.00)	154.50 (99.00–209.00)	0.059
HB (g/dL)	11.80 (10.50–12.90)	11.60 (9.90–12.90)	0.002	9.30 (7.90–11.30)	8.45 (6.10–11.10)	0.042
RBC (m/uL)	3.87 (3.41–4.29)	3.75 (3.27–4.25)	<0.001	3.10 (2.50–3.80)	2.70 (2.10–3.60)	0.050
WBC (k/uL)	10.10 (7.50–13.30)	11.00 (8.10–14.40)	<0.001	12.00 (8.70–15.80)	14.15 (10.00–20.00)	0.016
Scr (mg/dL)	0.80 (0.70–0.90)	0.80 (0.70–1.10)	<0.001	0.77 (0.62–0.94)	1.56 (1.18–2.13)	<0.001
BUN (mg/dL)	13.00 (10.00–18.00)	15.00 (11.00–21.00)	<0.001	15.12 (10.92–19.3)	26.60 (18.48–39.20)	<0.001
PT (s)	12.60 (11.70–13.70)	13.00 (11.90–14.60)	<0.001	14.00 (13.60–15.00)	16.00 (14.80–18.40)	<0.001
APTT (s)	27.80 (25.65–30.50)	27.70 (25.30–30.70)	0.018	36.00 (33.00–40.00)	41.00 (37.00–50.00)	<0.001
Chloride (mEq/L)	104.00 (101.00–107.00)	104.00 (102.00–108.00)	<0.001	109.00 (106.00–113.00)	113.50 (109.00–119.00)	0.141
Potassium (mEq/L)	4.00 (3.70–4.30)	4.00 (3.70–4.40)	0.011	3.50 (3.20–3.80)	3.70 (3.40–4.20)	0.002
Sodium (mEq/L)	139.00 (136.00–142.00)	139.00 (137.00–142.00)	0.067	139.00 (136.00–142.00)	142.50 (138.00–149.00)	<0.001
Glucose (mg/dL)	110.00 (96.00–131.00)	120.00 (103.00–143.00)	<0.001	136.80 (118.80–162.00)	142.20 (117.00–190.80)	0.146
Glucose measurements	6.00 (4.00–10.00)	11.00 (6.00–22.00)	<0.001	43.00 (36.00–43.00)	43.00 (43.00–43.00)	0.33
Measurement density	2.36 (1.63–3.98)	2.07 (1.47–3.25)	0.035	3.91 (2.53–4.78)	3.07 (2.05–4.30)	0.037
GV	14.54 (10.07–19.00)	15.88 (12.41–20.40)	<0.001	15.59 (13.55–19.06)	19.69 (15.41–25.76)	<0.001
Comorbidities						
Sepsis (%)	274.00 (31.14%)	758.00 (59.64%)	<0.001	27.00 (15.43%)	33.00 (36.67%)	<0.001
Hypertension (%)	402.00 (45.68%)	595.00 (46.81%)	0.605	21.00 (12.00%)	19.00 (21.11%)	0.050
Diabetes (%)	119.00 (13.52%)	222.00 (17.47%)	0.014	20.00 (11.43%)	10.00 (11.11%)	0.938
CHF (%)	60.00 (6.82%)	142.00 (11.17%)	<0.001	13.00 (7.43%)	20.00 (22.22%)	<0.001
COPD (%)	61.00 (6.93%)	79.00 (6.22%)	0.508	9.00 (5.14%)	11.00 (12.22%)	0.039
Liver disease (%)	55.00 (6.25%)	107.00 (8.42%)	0.061	29.00 (16.57%)	16.00 (17.78%)	0.804
Intervention						
Vasoactive drugs (%)	55.00 (6.25%)	205.00 (16.13%)	<0.001	59.00 (33.71%)	53.00 (58.89%)	<0.001
CRRT (%)	1.00 (0.11%)	21.00 (1.65%)	<0.001	1.00 (0.57%)	30.00 (33.33%)	<0.001
Ventilator use (%)	512.00 (58.18%)	1,074.00 (84.50%)	<0.001	148.00 (84.57%)	87.00 (96.67%)	0.003
Outcomes						
In-ICU 28 day mortality (%)	32.00 (3.64%)	178.00 (14.00%)	<0.001	4.00 (2.29%)	31.00 (34.44%)	<0.001

SBP, systolic blood pressure; MBP, mean blood pressure; PLT, platelet count; HB, hemoglobin; RBC, red blood cells; WBC, white blood cell; Scr, serum creatinine; BUN, blood urea nitrogen; PT, prothrombin time; APTT, activated partial thromboplastin time; GV, glycemic variability; Measurement density, the number of glucose measurements divided by ICU length of stay (measurements per ICU day); CHF, chronic heart failure; COPD, chronic obstructive pulmonary disease; AKI, acute kidney injury; CRRT, continuous renal replacement therapy.

The independent cohort, which included 265 TBI patients, showed similar patterns. In this cohort, patients with AKI (*n* = 90) had significantly higher GV compared with those without AKI (GV: 19.69 vs. 15.59, *p* < 0.001). Detailed baseline characteristics of both cohorts are summarized in Table 1.

### Relationship between GV and AKI risk in MIMIC IV database

Univariate logistic regression analysis was first performed to identify variables associated with AKI in patients with TBI (*p* < 0.05). These candidate variables were subsequently entered into multivariable logistic regression models to adjust for potential confounders. After adjustment for these covariates—including hemoglobin (HB), red blood cell count (RBC), platelet count (PLT), WBC, PT, APTT, Scr, BUN, blood glucose, glucose measurements, measurement density, potassium, chloride, heart rate, sepsis, chronic heart failure (CHF), diabetes, APACHE II score, SOFA score, and use of vasoactive drugs—GV remained a risk factor for AKI (OR = 1.02; 95% CI 1.01–1.03; *p* = 0.004). Detailed results are presented in Table 2.

**Table 2:** Univariate and multivariate logistic regression analysis of incidence rate of AKI in patients with TBI in the MIMIC database.

Variables	Univariate model		Multivariable model	
	*P*-value	OR (95%CI)	*P*-value	OR (95%CI)
Age	0.103	1 (0.99–1.01)		
Gender (Male)	0.057	1.19 (1.00–1.42)		
Heart rate	0.009	1.01 (1.00–1.01)	0.996	1 (0.99–1.01)
Temperature	0.347	0.97 (0.89–1.03)		
SBP	0.375	1.01 (1.00–1.01)		
MBP	0.493	1 (0.99–1.00)		
SPO_2_	0.23	1.02 (1.00–1.04)		
SOFA	<0.001	1.25 (1.20–1.31)	0.011	1.08 (1.02–1.14)
GCS	0.272	0.98 (0.9–1.01)		
APACHE II	<0.001	1.08 (1.06–1.10)	0.048	1.02 (1.00–1.05)
PLT	0.025	1 (0.99–1.00)	0.985	1 (1.00–1.00)
HB	0.002	0.94 (0.90–0.98)	0.144	1.08 (0.97–1.20)
RBC	<0.001	0.81 (0.71–0.92)	0.314	0.85 (0.63–1.16)
WBC	<0.001	1.03 (1.01–1.05)	0.401	1.01 (0.99–1.03)
Scr	<0.001	2.13 (1.66–2.80)	0.059	1.28 (1.01–1.69)
BUN	<0.001	1.04 (1.03–1.05)	0.094	1.01 (1.00–1.03)
PT	<0.001	1.1 (1.06–1.14)	0.157	1.03 (0.99–1.07)
APTT	0.028	1.01 (1.00–1.02)	0.925	1 (0.99–1.01)
Chloride	0.001	1.03 (1.01–1.04)	0.04	1.02 (1.00–1.04)
Potassium	0.015	1.18 (1.03–1.34)	0.302	1.08 (0.93–1.25)
Sodium	0.068	1.02 (1.00–1.04)		
Glucose	<0.001	1.01 (1.00–1.01)	<0.001	1.01 (1.00–1.01)
GV	<0.001	1.04 (1.03–1.05)	0.004	1.02 (1.01–1.03)
Glucose measurements	<0.001	1.05 (1.04–1.06)	<0.001	1.07 (1.06–1.09)
Measurement density	0.039	0.98 (0.96–0.99)	<0.001	0.85 (0.82–0.88)
Sepsis	<0.001	3.27 (2.73–3.92)	<0.001	2.44 (2.00–2.98)
CHF	0.001	1.72 (1.26–2.37)	0.068	1.39 (0.98–2.00)
COPD	0.508	0.89 (0.63–1.26)		
Hypertension	0.605	1.05 (0.88–1.24)		
Diabetes	0.014	1.35 (1.07–1.73)	0.614	1.08 (0.81–1.43)
Liver disease	0.062	1.38 (0.99–1.94)		
Vasoactive drugs	<0.001	2.88 (2.13–3.97)	0.006	1.61 (1.15–2.28)

SBP, systolic blood pressure; MBP, mean blood pressure; PLT, platelet count; HB, hemoglobin; RBC, red blood cells; WBC, white blood cell; Scr, serum creatinine; BUN, blood urea nitrogen; PT, prothrombin time; APTT, activated partial thromboplastin time; GV, glycemic variability; Measurement density, the number of glucose measurements divided by ICU length of stay (measurements per ICU day); CHF, chronic heart failure; COPD, chronic obstructive pulmonary disease.

**Figure 2:**
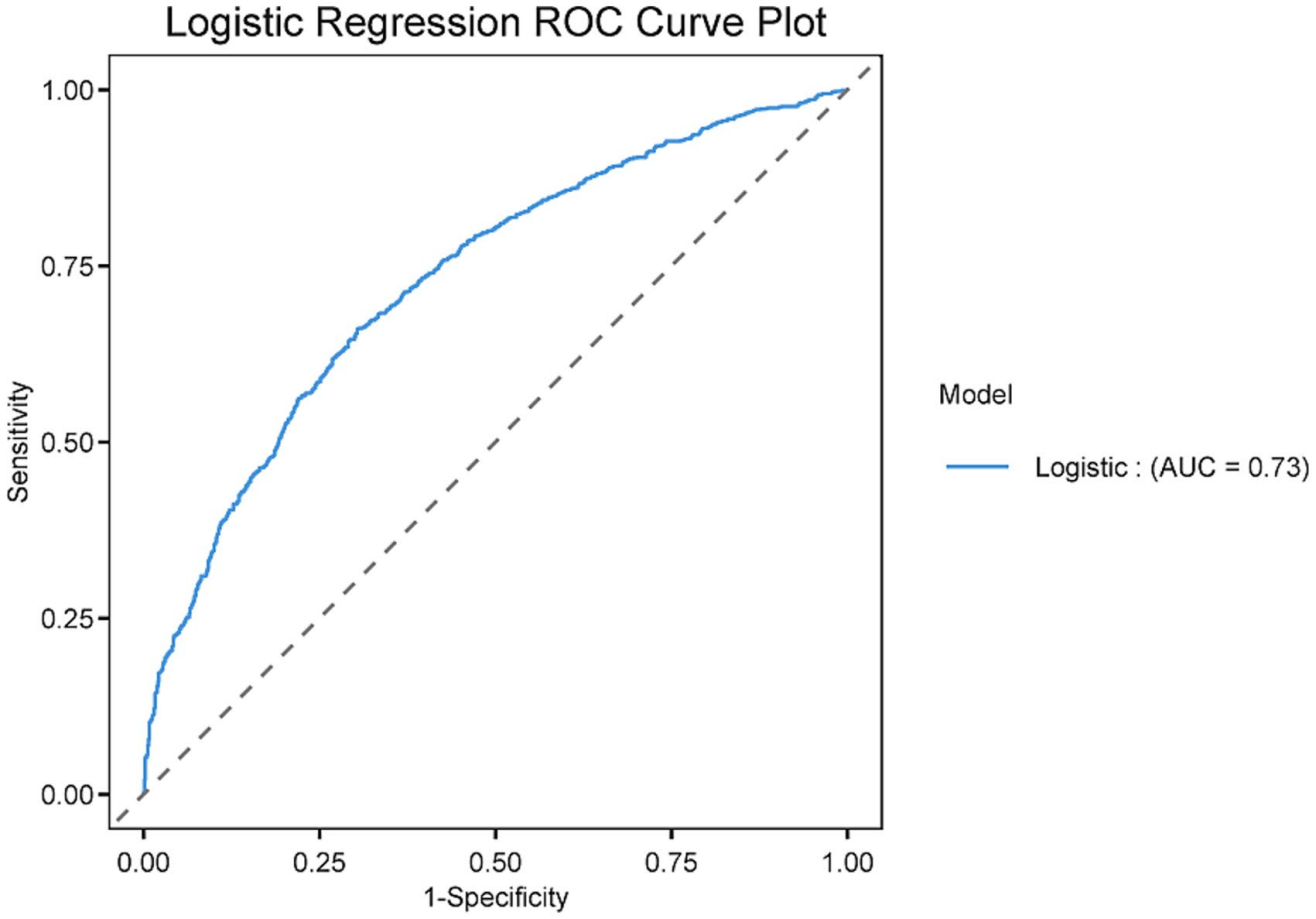
ROC curve of GV for prediction of AKI after adjustment for all variables in MIMIC IV database.

To further assess the predictive performance of GV, ROC curves were constructed. After adjustment for all variables, the AUC was 0.73 (95% CI: 0.71–0.75), sensitivity of 66.0%, and specificity of 69.7% (Figure 2). We then developed three models to examine the association between GV and AKI after adjustment for confounding factors (Table 3). A robust positive relationship was observed. In the unadjusted model, higher GV was associated with an increased risk of AKI (Model 1: OR = 1.46; 95% CI: 1.29–1.67). This association remained statistically significant in both the partially adjusted and fully adjusted models (Model 2: OR = 1.18; 95% CI: 1.03–1.35; Model 3: OR = 1.16; 95% CI: 1.02–1.34). An optimal cutoff value of 12.02 for GV was determined using ROC curve analysis (Supplementary Figure 2A). GV was subsequently categorized according to the optimal cutoff value for additional analysis. As shown in Table 3, the positive association between higher GV and AKI risk persisted across all models (Model 1: OR = 2.31; 95% CI: 1.91–2.80; Model 2: OR = 1.54; 95% CI: 1.25–1.90; Model 3: OR = 1.52; 95% CI: 1.23–1.88). In conclusion, GV provides some predictive value for the occurrence of AKI in patients with TBI. In addition, we plotted clinical decision curves to assess the improved clinical utility of the GV. The results showed that the net clinical benefit of each scoring tool also improved after considering the GV (Supplementary Figure 3). In addition, when considering the GV, we also calculated the IDI as well as the NRI of the scoring tools (SOFA and APACHE II) to analyze the effect of the GV on the predictive ability of the scoring tools. The IDI and the NRI are both tools for assessing the degree of improvement in the predictive ability of a response model, with greater than 0 indicating a positive improvement, and less than 0 indicating a negative improvement. The predictive ability of the scoring tool (specially SOFA) for the incidence of AKI in patients with traumatic brain injury was significantly improved (*p* < 0.05) after considering the GV (Supplementary Table 3). Sensitivity analyses restricted to patients with ≥5 glucose measurements yielded results consistent with the primary analysis, indicating that the association was not driven by sparse sampling. Substitution of glucose SD for CV in multivariable models produced comparable effect estimates, suggesting that the observed relationship between GV and AKI was robust across variability definitions (Supplementary Table 4).

**Table 3:** Logistic regression model for the association of GV with AKI incidence in the MIMIC database and independent cohort.

GV	Model 1		Model 2		Model 3	
	OR (95%CI)	*P*-value	OR (95%CI)	*P*-value	OR (95%CI)	*P*-value
Analysis Cohort						
Continuous variable per 10 unit	1.46 (1.29–1.67)	<0.001	1.18 (1.03–1.35)	0.026	1.16 (1.02–1.34)	0.034
Categories						
<12.02	Ref.		Ref.		Ref.	
≥12.02	2.31 (1.91–2.80)	<0.001	1.54 (1.25–1.90)	<0.001	1.52 (1.23–1.88)	<0.001
Independent cohort						
Continuous variable per 10 unit	2.91 (1.92–4.56)	<0.001	6.78 (2.02–31.32)	0.005	9.25 (1.70–97.94)	0.031
Categories						
<17.08	Ref.		Ref.		Ref.	
≥17.08	3.56 (3.10–6.12)	<0.001	8.65 (1.98–46.75)	0.006	6.40 (2.24–20.33)	<0.001

Model 1: Unadjusted. Model 2: Adjusted for variables that were statistically different between the two groups; baseline glucose level and history of diabetes mellitus were additionally included. Model 3: Adjusted for all.

**Figure 3:**
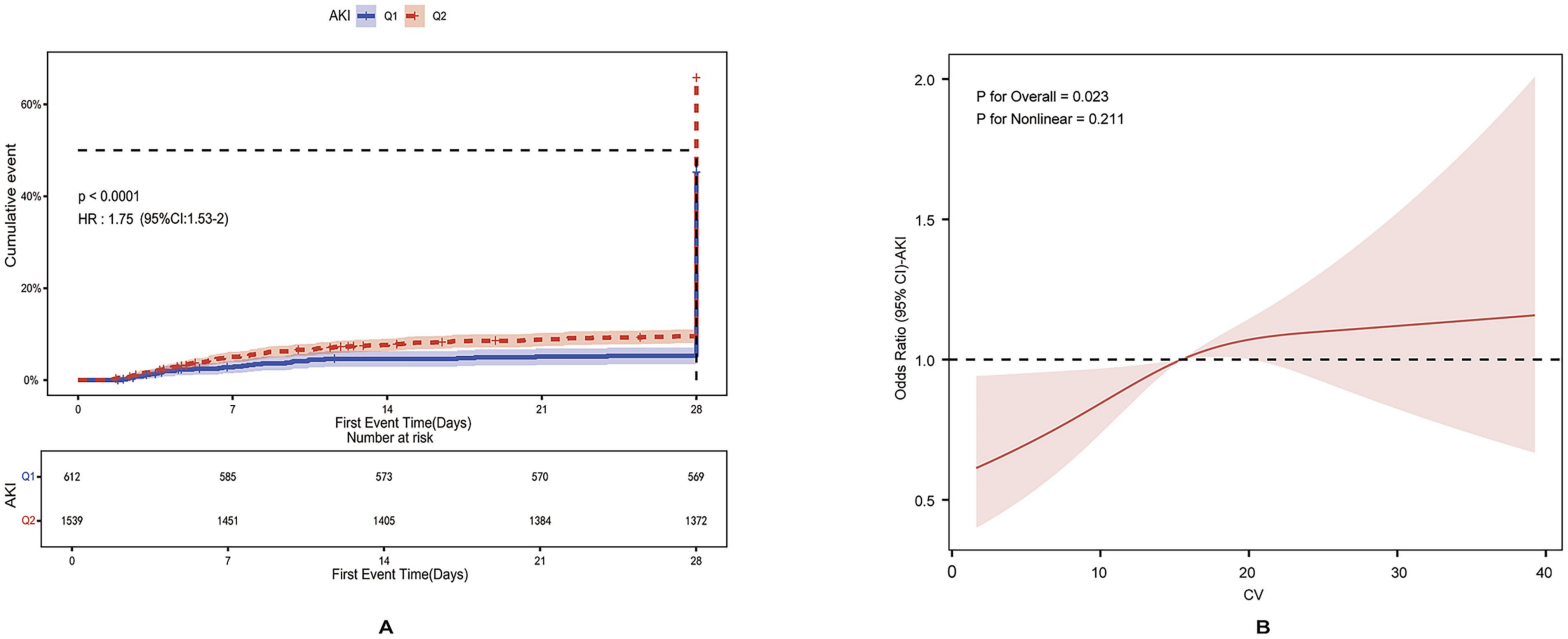
**(A)** Cumulative event incidence curves for incidence of AKI in MIMIC IV database. **(B)** RCS dose–response relationship between AKI and GV in MIMIC IV database.

We divided the patients into low- and high-GV groups based on the optimal cutoff value. Cumulative incidence curves showed that patients in the high-GV group (CV ≥ 12.02) had a significantly higher incidence of AKI than those in the low-GV group (*p* < 0.001) (Figure 3A). To further characterize this relationship, a RCS model was applied. As shown in Figure 3B, there was a dose–response relationship between GV and AKI risk (*p* = 0.21 for Nonlinear, *p* = 0.023 for Overall), supporting a positive association between increasing GV and higher AKI risk. In addition, Supplementary Figure 5 shows that the 28-day survival rate in the ICU was significantly lower in the high-GV group (CV ≥ 12.02) than in the low-GV group (CV < 12.02) (log-rank *p* < 0.05). These findings suggest that elevated GV is strongly associated not only with an increased risk of AKI but also with reduced short-term (28-day) survival in patients with TBI.

### Influence of glucose monitoring intensity on the association between glycemic variability and AKI

To examine whether differences in glucose monitoring intensity influenced the observed association between GV and AKI, additional analyses incorporating measures of monitoring frequency were performed. As shown in Table 4, GV remained significantly associated with AKI across all models. In the fully adjusted model, each one-unit increase in GV was associated with a higher risk of AKI (OR 1.02, 95% CI 1.01–1.04; *p* = 0.007). Further adjustment for the total number of glucose measurements (Model 2^b^) or for measurement density (Model 3^c^) yielded similar effect estimates, indicating that the GV–AKI association was robust to differences in glucose monitoring frequency.

**Table 4:** Association between glycemic variability and AKI after adjustment for monitoring intensity.

Variables	Model 1^a^		Model 2^b^		Model 3^c^	
	OR (95%CI)	*P*-value	OR (95%CI)	*P*-value	OR (95%CI)	*P*-value
GV (continuous variable per 1 unit)	1.02 (1.01–1.04)	0.007	1.02 (1.01–1.03)	0.034	1.02 (1.01–1.04)	0.003

Model 1^a^: Fully adjusted model. Model 2^b^: Model 1^a^ additionally adjusted for the number of glucose measurements. Model 3^c^: Model 1^a^ additionally adjusted for measurement density.

Sensitivity analyses stratified by tertiles of glucose monitoring density demonstrated consistent results across all strata (Table 5). In the low monitoring density group (<1.718 measurements per ICU day), GV was significantly associated with AKI (OR 1.08, 95% CI 1.05–1.11; *p* < 0.001). Comparable associations were observed in the medium monitoring density group (OR 1.04, 95% CI 1.02–1.06; *p* < 0.001) and in the high monitoring density group (OR 1.03, 95% CI 1.01–1.06; *p* = 0.008).

**Table 5:** Association between glycemic variability and AKI across tertiles of glucose monitoring density.

Measurement density tertiles	N	AKI (%)	GV median (IQR)	OR (GV)	95%CI	*p*-value
Low (<1.718)	717	65.7	14.34 (10.05–18.74)	1.08	1.05–1.11	<0.001
Medium (1.718–2.941)	718	58.5	15.90 (11.53–19.84)	1.04	1.02–1.06	<0.001
High (>2.941)	716	53.1	16.01 (12.39–20.40)	1.03	1.01–1.06	0.008

Measurement density, the number of glucose measurements divided by ICU length of stay (measurements per ICU day). Patients were categorized into tertiles of monitoring density representing low, medium, and high monitoring intensity. Odds ratios (ORs) with 95% confidence intervals (CIs) were estimated from multivariable logistic regression models with GV entered as a continuous variable. GV values are reported as median (interquartile range, IQR).

Finally, as shown in Supplementary Table 5, formal interaction testing between GV and measurement density did not demonstrate a statistically significant interaction (*P* for interaction = 0.73), suggesting that the association between GV and AKI was not materially modified by differences in glucose monitoring intensity.

### Subgroup analyses in MIMIC IV database

**Figure 4:**
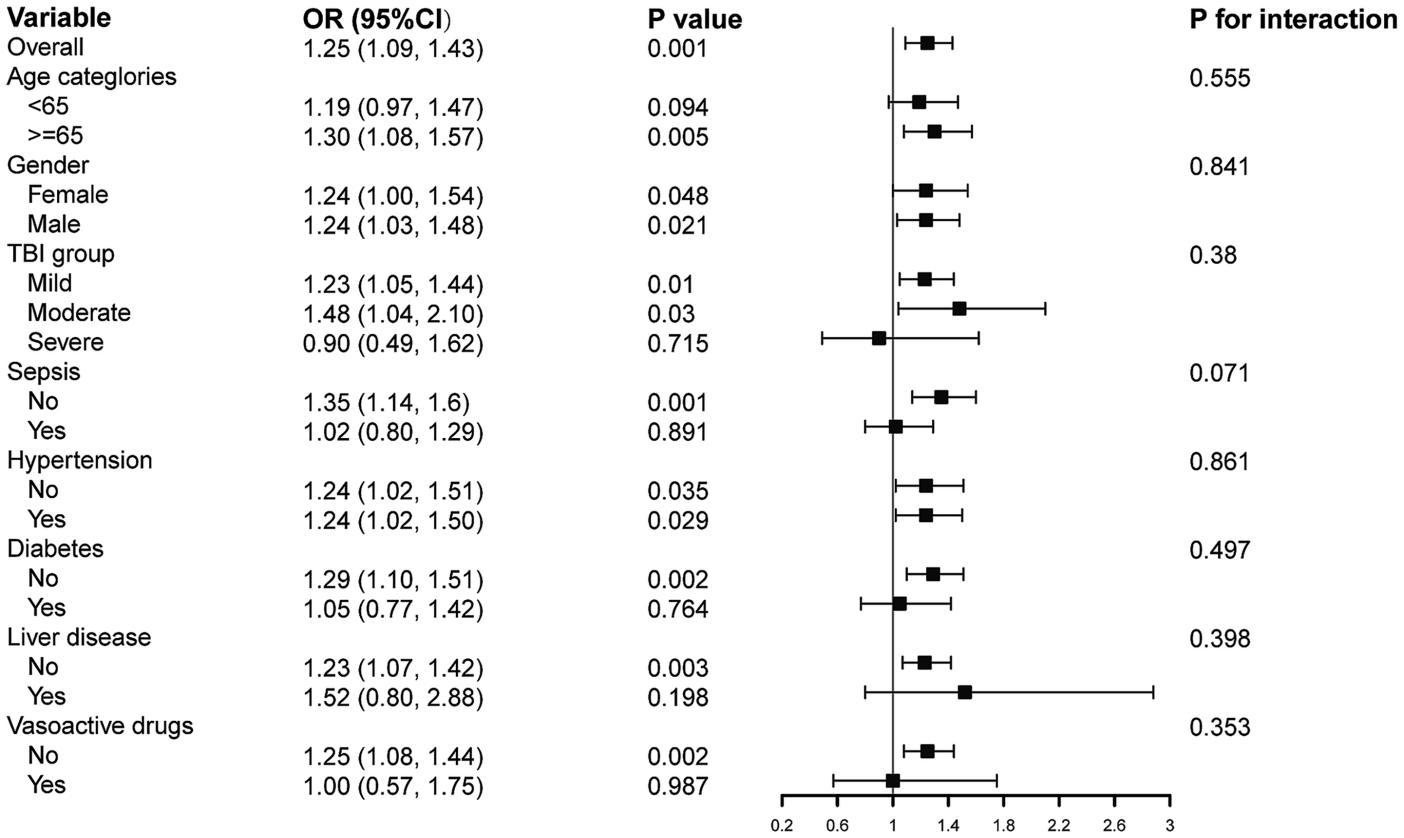
Subgroup analyses for the association of GV with AKI in MIMIC IV database. OR, Odds ratio; CI, Confidence interval.

Subgroup analyses were performed to evaluate whether the association between GV and AKI differed across specific patient characteristics, including age, gender, TBI groups, sepsis, diabetes mellitus, hypertension, history of chronic liver disease, and use of vasoactive drugs (Figure 4). The analysis found that higher GV was consistently associated with an increased risk of AKI. Moreover, no significant interactions were detected, indicating that the relationship between GV and AKI did not meaningfully vary by subgroup. These findings suggest that the association between elevated GV and increased AKI risk is robust and remains stable across the majority of the patient population.

### Relationship between GV and AKI risk in the independent cohort

Independent Cohort Analysis was performed using data from 265 TBI patients obtained from the electronic medical record system of the Affiliated Hospital of Guangdong Medical University (Table 1). As shown in Supplementary Table 6, after adjustment for a wide range of variables—including gender, age, heart rate, systolic blood pressure (SBP), mean blood pressure (MBP), BUN, Scr, potassium, sodium, chloride, WBC, RBC, HB, PT, APTT, measurement density, sepsis, CHF, chronic obstructive pulmonary disease (COPD), APACHE II score, GCS score, SOFA score, and use of vasoactive drugs—GV remained a risk factor for AKI (OR = 1.25; 95% CI 1.07–1.53, *p* = 0.015).

To further evaluate the predictive ability of GV for AKI in the independent cohort, after multivariable adjustment, GV remained significantly associated with increased AKI risk (highest vs. lowest GV group: OR = 6.40; 95% CI: 2.24–20.33; *p* < 0.001) (Table 3). When treated as a continuous variable, higher GV was likewise associated with a greater incidence of AKI (OR = 9.25; 95% CI: 1.70–97.94; *p* = 0.031) (Table 3).

**Figure 5:**
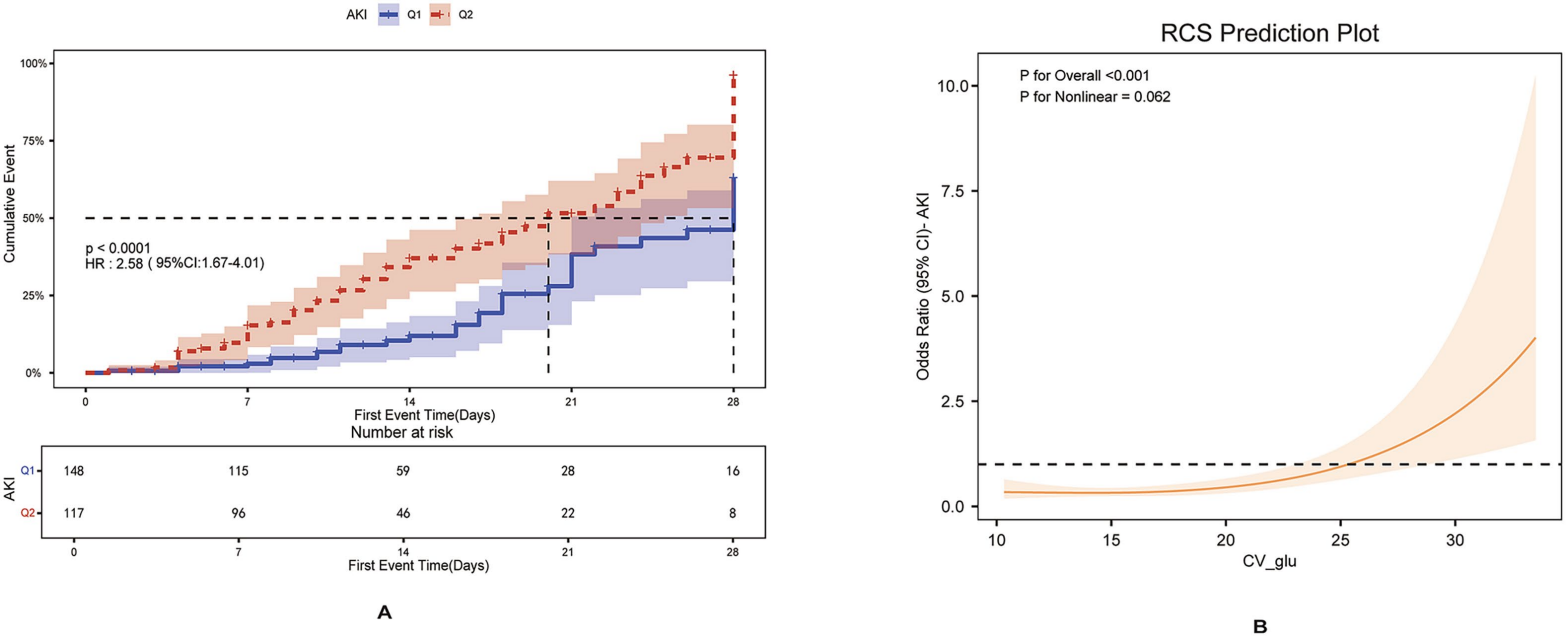
**(A)** Cumulative event incidence curves for incidence of AKI in the independent cohort. **(B)** RCS dose–response relationship between AKI and GV in the independent cohort.

An optimal cutoff value of 17.08 for GV was determined using ROC curve analysis (Supplementary Figure 2B) and applied to classify patients into low- and high-GV groups. As shown in Figure 5A, the cumulative risk of AKI was significantly higher in the high-GV group (*p* < 0.001). Figure 5B, based on the RCS model, further supports a linear dose–response relationship between GV and AKI risk (*p* = 0.062 for non-linearity; *p* < 0.001 for overall). Supplementary Figure 6 demonstrates that elevated GV is also associated with significantly lower 28-day ICU survival, with a statistically significant difference between groups (log-rank *p* < 0.001).

### Subgroup analyses in the independent cohort

**Figure 6:**
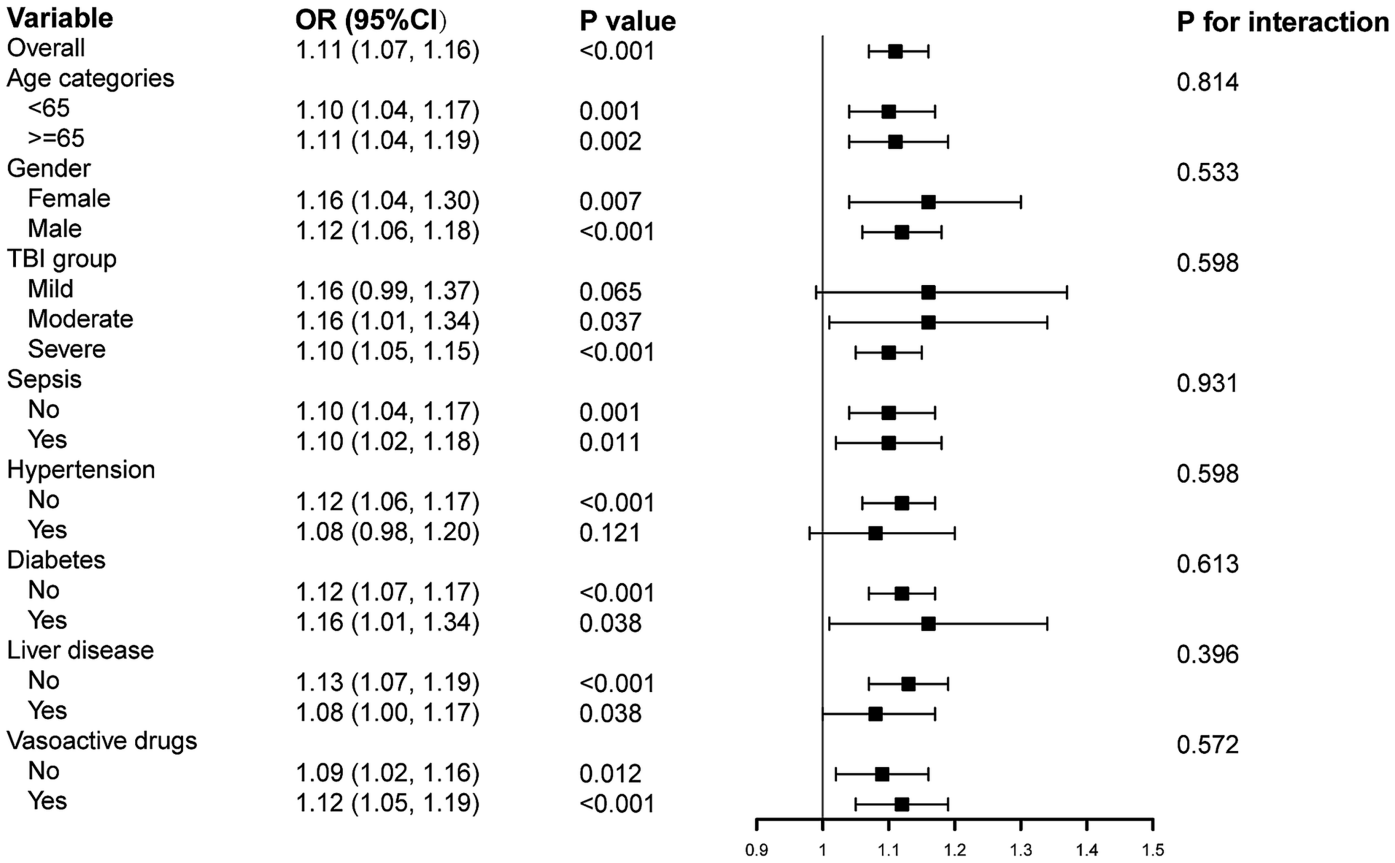
Subgroup analyses for the association of GV with AKI in the independent cohort.

Subgroup analysis (Figure 6) shows that, consistent with the findings in the primary cohort, GV remains an independent risk factor for AKI among TBI patients in the independent cohort, with a positive association observed across most subgroups.

## Discussion

This retrospective cohort study, complemented by an independent cohort replication, evaluated the relationship between GV and AKI in critically ill patients with TBI. Higher GV, quantified using the CV, was associated with increased odds of AKI. The association persisted after adjustment for demographic characteristics, comorbidities, and illness severity. Restricted cubic spline analysis further demonstrated a progressive and approximately linear increase in AKI risk with increasing GV levels. Importantly, similar patterns observed in the independent cohort support the reproducibility of this relationship and suggest that it is unlikely to be confined to a single dataset. These findings are broadly consistent with previous ICU studies linking elevated GV to adverse outcomes and organ dysfunction (16, 10).

In critically ill patients, glycemic instability reflects more than deviations in mean glucose levels. Variability-based indices such as CV and SD capture dynamic glucose fluctuations that may signal underlying physiological stress not apparent from static measurements alone (9, 16). CV was selected as the primary metric because it expresses variability relative to mean glucose levels, allowing comparisons across individuals with different baseline glycemic states. Analyses based on SD yielded comparable results, indicating that the observed association was not dependent on a single definition of variability. This consistency aligns with methodological recommendations encouraging evaluation of multiple GV indices whenever feasible (21).

The biological plausibility of this association is supported by the pathophysiological context of acute brain injury. Severe TBI induces neuroendocrine activation and systemic inflammatory responses that may compromise renal perfusion and microvascular integrity (5, 15). In parallel, glucose oscillations have been linked to oxidative stress and endothelial dysfunction—mechanisms that may heighten susceptibility to organ injury during critical illness (25, 33). Within neurocritical care environments, where metabolic stress is often pronounced, such fluctuations may further destabilize renal homeostasis.

Subgroup analyses were prespecified on clinical and biological grounds, including age, gender, baseline neurological severity, and comorbid conditions relevant to metabolic and vascular regulation. These variables were selected because they may influence glucose homeostasis, systemic stress responses, and susceptibility to organ injury, and therefore could plausibly modify the association between GV and AKI. Baseline neurological severity may reflect the degree of neuroendocrine stress and metabolic instability in critically ill patients, whereas comorbid conditions such as diabetes, sepsis, hypertension, and chronic liver disease are closely linked to altered glucose metabolism, vascular dysfunction, and renal vulnerability. Despite these potential modifiers, little evidence of interaction was observed across most strata, indicating that the GV–AKI association was broadly consistent across clinically heterogeneous patient groups. Nevertheless, several subgroup patterns merit consideration. The association appeared more evident among non-septic patients, possibly reflecting differences between sepsis-related inflammatory mechanisms and the autonomic dysregulation that predominates after TBI (34). Similarly, no significant association was observed among patients with pre-existing diabetes, whereas non-diabetic individuals appeared more susceptible to the adverse effects of GV, a pattern consistent with previous reports (35). Taken together, these observations support the robustness of the association while suggesting that its magnitude may vary according to the underlying metabolic and inflammatory context.

Baseline glucose level and history of diabetes mellitus were retained in all multivariable models regardless of their statistical significance in univariable analyses. Because GV is intrinsically related to overall glycemic exposure, this adjustment was necessary to distinguish fluctuation-related effects from sustained hyperglycemia. Variance inflation factors remained within acceptable limits, supporting stable parameter estimation and indicating no evidence of problematic multicollinearity (23).

GV was evaluated in this study as an adjunctive prognostic marker rather than as a standalone predictive instrument. Although the improvement in discrimination (ΔAUC) was modest, complementary analyses—including calibration assessment, reclassification indices, and decision curve analysis—demonstrated consistent directional improvements across several performance domains. Together, these findings suggest that incorporating GV into established severity scores may enhance risk stratification. This interpretation is consistent with current methodological guidance emphasizing multidimensional evaluation of incremental predictive value beyond AUC alone (25, 36–38).

An additional methodological consideration when evaluating GV in ICU populations is the potential influence of monitoring intensity. Patients with longer ICU stays often undergo more frequent glucose testing, which can inflate estimates of variability and introduce time-related bias. To address this issue, both the total number of glucose measurements and measurement density were incorporated into the adjusted analyses. Measurement density—defined as the number of glucose measurements per ICU day—provides a standardized indicator of monitoring intensity that accounts for differences in ICU length of stay. Compared with predefined thresholds based on measurement counts, stratification by measurement-density tertiles allows data-driven grouping and reduces subjectivity in defining monitoring intensity categories. In the present study, the association between GV and AKI remained consistent across monitoring-density strata, and formal interaction testing showed no evidence of significant effect modification. These findings suggest that the observed relationship between GV and AKI is unlikely to be explained solely by differences in glucose monitoring frequency. Nevertheless, as with all observational analyses based on routine clinical data, residual confounding related to monitoring practices cannot be completely excluded. Similar concerns regarding surveillance bias and exposure misclassification have been highlighted in prior ICU monitoring studies (39, 40).

Several limitations should be considered. First, the retrospective design limits causal inference and does not eliminate the possibility of residual confounding despite extensive multivariable adjustment. Second, although GV was calculated using glucose measurements recorded during the ICU stay until the occurrence of AKI or ICU discharge, exposure was not defined within a standardized admission-anchored time window (e.g., 0–24 or 0–48 h after ICU admission). Consequently, both the duration and frequency of glucose monitoring may have differed across patients, introducing heterogeneity in exposure assessment. Such variability may contribute to time-related bias, a recognized concern in observational analyses based on routinely collected clinical data (41, 42). Although additional adjustments for glucose measurement frequency and measurement density were performed to mitigate this issue, heterogeneity in monitoring practices cannot be entirely excluded, and some degree of between-patient incomparability may still persist. Finally, although the independent cohort supported the primary findings, it was derived from a single center with a relatively modest sample size, which may limit the generalizability of the results.

The analysis conducted in the independent cohort should therefore not be interpreted as formal external validation. In this study, the optimal cut-off derived from the development cohort was not directly applied to the independent dataset, and the original model parameters were not preserved in a fixed (“frozen”) form. Instead, the optimal threshold was re-estimated within the independent cohort. According to established methodological standards for prediction modeling, including the TRIPOD recommendations, this approach represents cohort-specific model updating or re-fitting rather than strict external validation (30, 31).

The difference in optimal cut-off values observed between the two cohorts (12.02 vs. 17.08) likely reflects differences in case mix, illness severity, glucose monitoring intensity, and outcome prevalence. Because receiver operating characteristic–based threshold selection is inherently sample-dependent, variability in optimal thresholds across datasets is not unexpected. At the same time, these cohort-specific differences highlight the context-dependent nature of biomarker calibration and emphasize the need for cautious interpretation before clinical implementation. Accordingly, the independent cohort findings should be interpreted as evidence of reproducibility and incremental prognostic utility rather than validation of a fixed predictive model. Future studies applying a prespecified and frozen model in independent populations will be required to achieve formal external validation (38, 41, 42).

Several avenues for future investigation emerge from these findings. Prospective studies incorporating predefined admission-anchored exposure windows would improve temporal standardization and strengthen causal interpretation, in line with recommendations for target-trial emulation in observational analyses (42). Continuous glucose monitoring (CGM) may further refine the characterization of glycemic dynamics. High-resolution CGM-derived metrics such as TIR and MAGE capture aspects of variability that intermittent measurements may overlook (11, 12, 42, 43). Mechanistic research is also needed to determine whether GV primarily reflects systemic instability or directly contributes to renal injury through oxidative and microvascular pathways. Broader multicenter validation will be necessary to evaluate calibration stability and determine clinically meaningful implementation thresholds. Finally, whether targeted reduction of glycemic variability can improve renal or neurological outcomes remains uncertain. Although mean glucose control remains central to ICU management, interventional studies are needed to determine whether stabilizing glucose fluctuations confers additional clinical benefit.

## Conclusion

In this retrospective cohort study of critically ill patients with traumatic brain injury, greater glycemic variability was consistently associated with an increased risk of acute kidney injury. This relationship remained robust after adjustment for demographic characteristics, comorbidities, illness severity, and differences in glucose monitoring intensity, and was reproducible in an independent cohort. These findings suggest that glycemic variability may capture clinically relevant metabolic instability not fully reflected by mean glucose levels alone.

Although glycemic variability should not be interpreted as a standalone predictive instrument, it may provide incremental prognostic information when considered alongside established severity scores in neurocritical care populations. Future prospective studies with standardized exposure windows and higher-resolution glucose monitoring are needed to clarify the mechanistic basis of this association and to determine whether targeted stabilization of glucose fluctuations can improve renal and neurological outcomes.

## Data Availability

The original contributions presented in the study are included in the article/Supplementary material, further inquiries can be directed to the corresponding authors.
