# Supplementary material for: Association between glycemic variability and the risk of acute kidney injury in patients with traumatic brain injury: a retrospective cohort study with independent cohort analysis
Source: Front Neurol. 2026 Apr 13;17:1797958. doi: 10.3389/fneur.2026.1797958 (PMC13114571; doi:10.3389/fneur.2026.1797958)
Supplement: Supplementary file 1 [file Data_Sheet_1.docx]

**Supplementary Material**

**1.Supplementary Table 1. Multicollinearity test.**

**2.Supplementary Table 2. Distribution of sample size and AKI incidence across predefined categories of glucose measurement frequency.**

**3.Supplementary Table 3.The incremental effect of the GV in the MIMIC database and independent cohort.**

**4.Supplementary Table 4. Logistic regression model for the association of GV^1^ (at least five glucose measurements) and SD of glucose with AKI incidence.**

**5.Supplementary Table 5. Interaction analysis between glycemic variability and measurement density for AKI.**

**6.Supplementary Table 6.Performance of independent cohort analysis for the univariate and multivariate logistic regression analysis of incidence rate of AKU in patients with TBI.**

**7.Supplementary Figure 1. Calibration plots (A) in MIMIC IV database (B) in the independent cohort.**

**8.Supplementary Figure 2. ROC curve of GV for prediction of AKI (A) in MIMIC IV database (B) in the independent cohort.**

**9.Supplementary Figure 3. Decision curve analysis of scoring tools (A) SOFA; (B) APACHE II with and without considering the GV in MIMIC IV database.**

**10.Supplementary Figure 4. Decision curve analysis of scoring tools (A) SOFA; (B) APACHE II with and without considering the GV in the independent cohort.**

**11.Supplementary Figure 5. Kaplan–Meier survival analysis curves for 28-day ICU survival in MIMIC IV database.**

**12.Supplementary Figure 6. Kaplan–Meier survival analysis curves for 28-day ICU survival in the independent cohort.**

**1.Supplementary Table 1.** Multicollinearity test.

| **Variables** | **VIF** | **colinearity** |
| --- | --- | --- |
| Age | 2.191 | 0 |
| Gender (Male) | 1.252 | 0 |
| Heart rate | 1.187 | 0 |
| Temperature | 1.081 | 0 |
| SBP | 2.987 | 0 |
| MBP | 2.966 | 0 |
| SPO₂ | 1.130 | 0 |
| SOFA | 2.024 | 0 |
| GCS | 1.688 | 0 |
| APACHE II | 2.633 | 0 |
| PLT | 1.279 | 0 |
| HB | 5.359 | 0 |
| RBC | 5.179 | 0 |
| WBC | 1.235 | 0 |
| Scr | 1.560 | 0 |
| BUN | 1.801 | 0 |
| PT | 1.240 | 0 |
| APTT | 1.181 | 0 |
| Chloride | 2.793 | 0 |
| Potassium | 1.132 | 0 |
| Sodium | 2.605 | 0 |
| Glucose | 1.198 | 0 |
| GV | 1.057 | 0 |
| Glucose measurements | 1.966 | 0 |
| Measurement density | 1.898 | 0 |
| Sepsis | 1.162 | 0 |
| CHF | 1.197 | 0 |
| COPD | 1.120 | 0 |
| Hypertension | 1.361 | 0 |
| Diabetes | 1.259 | 0 |
| Liver disease | 1.139 | 0 |
| Vasoactive drugs | 1.117 | 0 |

SBP, systolic blood pressure; MBP, mean blood pressure; PLT, platelet count; HB, hemoglobin; RBC, red blood cells; WBC, white blood cell; Scr, serum creatinine; BUN, blood urea nitrogen; PT, prothrombin time; APTT, activated partial thromboplastin time; GV, glycaemic variability; Measurement density, the number of glucose measurements divided by ICU length of stay (measurements per ICU day); CHF, chronic heart failure; COPD, chronic obstructive pulmonary disease.

**2.Supplementary Table 2.Distribution of sample size and AKI incidence across predefined categories of glucose measurement frequency.**

| **Glucose measurements** | **N** | **AKI(%)** |
| --- | --- | --- |
| 5-7 | 988 | 44.4 |
| 8-10 | 292 | 60.3 |
| >10 | 871 | 75.3 |

Patients were categorized according to the total number of glucose measurements obtained during ICU stay (5-7, 8-10, and >10 measurements). The table shows the number of patients and the proportion of AKI events within each category. These data illustrate the imbalance in sample size across predefined measurement-count strata.

**3.Supplementary Table 3.** The incremental effect of the GV in the MIMIC database and independent cohort.

| **Score** | **AUC (95%CI)** | **AUC (95%CI)**  **(+GV)** | **P-value** | **IDI (95%CI)**  **(+GV)** | **P-value** | **NRI (95%CI)**  **(+GV)** | **P-value** |
| --- | --- | --- | --- | --- | --- | --- | --- |
| **Analysis Cohort** | | | | | | | |
| SOFA | 0.623 (0.605, 0.651) | 0.642 (0.620, 0.666) | 0.008 | 0.0092 (0.0051, 0.0132) | <0.001 | 0.1121 (0.0266, 0.1977) | 0.010 |
| APACHE II | 0.619 (0.595, 0.642) | 0.630 (0.607, 0.654) | 0.026 | 0.0077 (0.0039, 0.0114) | <0.001 | 0.08 (-0.0057, 0.1656) | 0.067 |
| **Independent Cohort** | | | | | | | |
| SOFA | 0.811 (0.758, 0.863) | 0.823 (0.771, 0.875) | 0.316 | 0.0446 (0.0168, 0.0724) | 0.002 | 0.4603 (0.2128, 0.7078) | <0.001 |
| APACHE II | 0.800 (0.740, 0.859) | 0.816 (0.757, 0.875) | 0.181 | 0.0370 (0.0115, 0.0625) | 0.005 | 0.4502 (0.2033, 0.6970) | <0.001 |

IDI, Integrated Discrimination Improvement; NRI, Net Reclassification Improvement.

**4.Supplementary Table 4.** Logistic regression model for the association of GV^1^ (at least five glucose measurements) and SD of glucose with AKI incidence.

| **Variables** | **Model 1** | | **Model 2** | | **Model 3** | |
| --- | --- | --- | --- | --- | --- | --- |
|  | **OR (95%CI)** | **P-value** | **OR (95%CI)** | **P-value** | **OR (95%CI)** | **P-value** |
| **GV^1^** (Continuous variable per 1 unit) | 1.03 (1.02-1.04) | <0.001 | 1.02 (1.01-1.03) | 0.044 | 1.01 (1.00-1.03) | 0.072 |
| **SD** (Continuous variable per 1 unit) | 1.04 (1.03-1.05) | <0.001 | 1.02 (1.01-1.03) | 0.005 | 1.02 (1.01-1.03) | 0.012 |

Model 1: Unadjusted.

Model 2: Adjusted for variables that were statistically different between the two groups; baseline glucose level and history of diabetes mellitus were additionally included.

Model 3: Adjusted for all.

**5.Supplementary Table 5.** Interaction analysis between glycemic variability and measurement density for AKI.

| **Variable** | **OR** | **95% CI** | **P-value** |
| --- | --- | --- | --- |
| Glycemic variability (per unit increase) | 1.02 | 1.01–1.04 | 0.026 |
| Measurement density (per ICU day) | 0.86 | 0.78-0.94 | 0.002 |
| GV × measurement density | 0.99 | 0.99–1.00 | 0.73 |

Odds ratios were estimated from logistic regression models including GV, measurement density, and their interaction term. Measurement density, the number of glucose measurements divided by ICU length of stay (measurements per ICU day). The interaction term (GV × measurement density) was used to evaluate whether the association between GV and AKI differed according to monitoring intensity.

**6.Supplementary Table 6.** Performance of independent cohort analysis for the univariate and multivariate logistic regression analysis of incidence rate of AKI in patients with TBI.

| **Variables** | **Univariate Model** | | **Multivariable Model** | |
| --- | --- | --- | --- | --- |
|  | **P-value** | **OR (95%CI)** | **P-value** | **OR (95%CI)** |
| Age | 0.005 | 1.02 (1.01-1.04) | 0.102 | 1.05 (0.99-1.10) |
| Gender (Male) | 0.004 | 3.24 (1.52-7.75) | 0.832 | 126 (0.16-13.10 ) |
| Heart rate | <0.001 | 1.02 (1.01-1.03) | 0.998 | 1.00 (0.97 -1.03 ) |
| Temperature | 0.130 | 1.27 (0.98-1.75) |  |  |
| SBP | 0.040 | 0.99 (0.98-1.00) | 0.597 | 1.01 (0.97 -1.06 ) |
| MBP | 0.026 | 0.98 (0.97-1.00 ) | 0.749 | 0.99 (0.93-1.06) |
| SPO₂ | 0.640 | 0.97 (0.88-1.09) |  |  |
| SOFA | <0.001 | 1.46 (1.32-1.63 ) | 0.175 | 1.22 (0.92-1.66 ) |
| GCS | <0.001 | 0.79 (0.72 -0.87 ) | 0.677 | 0.95 (0.72-1.23) |
| APACHEII | <0.001 | 1.19 (1.14-1.25 ) | 0.571 | 0.96 (0.83-1.11 ) |
| PLT | 0.052 | 1.00 (0.99-1.00) |  |  |
| HB | 0.031 | 0.99 (0.98-1.00 ) | 0.725 | 1.02 (0.93-1.13 ) |
| RBC | 0.033 | 0.74 (0.56-0.97 ) | 0.986 | 1.03 (0.05-18.85 ) |
| WBC | 0.030 | 1.04 (1.01-1.08 ) | 0.061 | 1.07 (0.98- 1.15 ) |
| Scr | <0.001 | 1.08 (1.06-1.11 ) | <0.001 | 1.12 (1.07-1.18) |
| BUN | <0.001 | 1.33 (1.23-1.46 ) | 0.272 | 0.90 (0.74 -1.08 ) |
| PT | <0.001 | 1.42 (1.25-1.64 ) | 0.569 | 1.08 (0.83-1.42) |
| APTT | <0.001 | 1.12 (1.08-1.16 ) | 0.308 | 1.06 (0.95-1.19) |
| Chloride | <0.001 | 1.13 (1.08-1.18) | 0.215 | 1.09 (1.01-1.30) |
| Potassium | 0.001 | 2.09 (1.37-3.29) | 0.817 | 1.14 (0.36-3.51) |
| Sodium | <0.001 | 1.11 (1.07-1.16) | 0.251 | 1.08 (0.93-1.22) |
| Glucose | 0.133 | 1.00 (1.00-1.01) |  |  |
| GV | <0.001 | 1.11 (1.07-1.16) | 0.015 | 1.25 (1.07-1.53) |
| Glucose measurements | 0.332 | 1.02 (0.98-1.05) |  |  |
| Measurement density | 0.026 | 0.82 (0.68-0.97) | 0.83 | 0.94 (0.53-1.67) |
| Sepsis | <0.001 | 3.17 (1.76-5.78) | 0.059 | 4.68 (0.98-25.40) |
| CHF | <0.001 | 3.56 (1.69-7.72) | 0.508 | 2.20 (0.21- 23.10) |
| COPD | 0.044 | 2.57 (1.02-6.61) | 0.948 | 0.91(0.43- 14.92 ) |
| Hypertension | 0.052 | 1.96 (0.99-3.89) |  |  |
| Diabetes | 0.938 | 0.97 (0.42-2.12) |  |  |
| Liver disease | 0.804 | 1.09 (0.55-2.11) |  |  |
| Vasoactive drugs | <0.001 | 2.82 (1.67-4.79) | 0.90 | 1.11 (0.20-5.92) |

SBP, systolic blood pressure; MBP, mean blood pressure; PLT, platelet count; HB, hemoglobin; RBC, red blood cells; WBC, white blood cell; Scr, serum creatinine; BUN, blood urea nitrogen; PT, prothrombin time; APTT, activated partial thromboplastin time; GV, glycaemic variability; Measurement density, the number of glucose measurements divided by ICU length of stay (measurements per ICU day); CHF, chronic heart failure; COPD, chronic obstructive pulmonary disease.

**7.Supplementary Figure 1.**

| 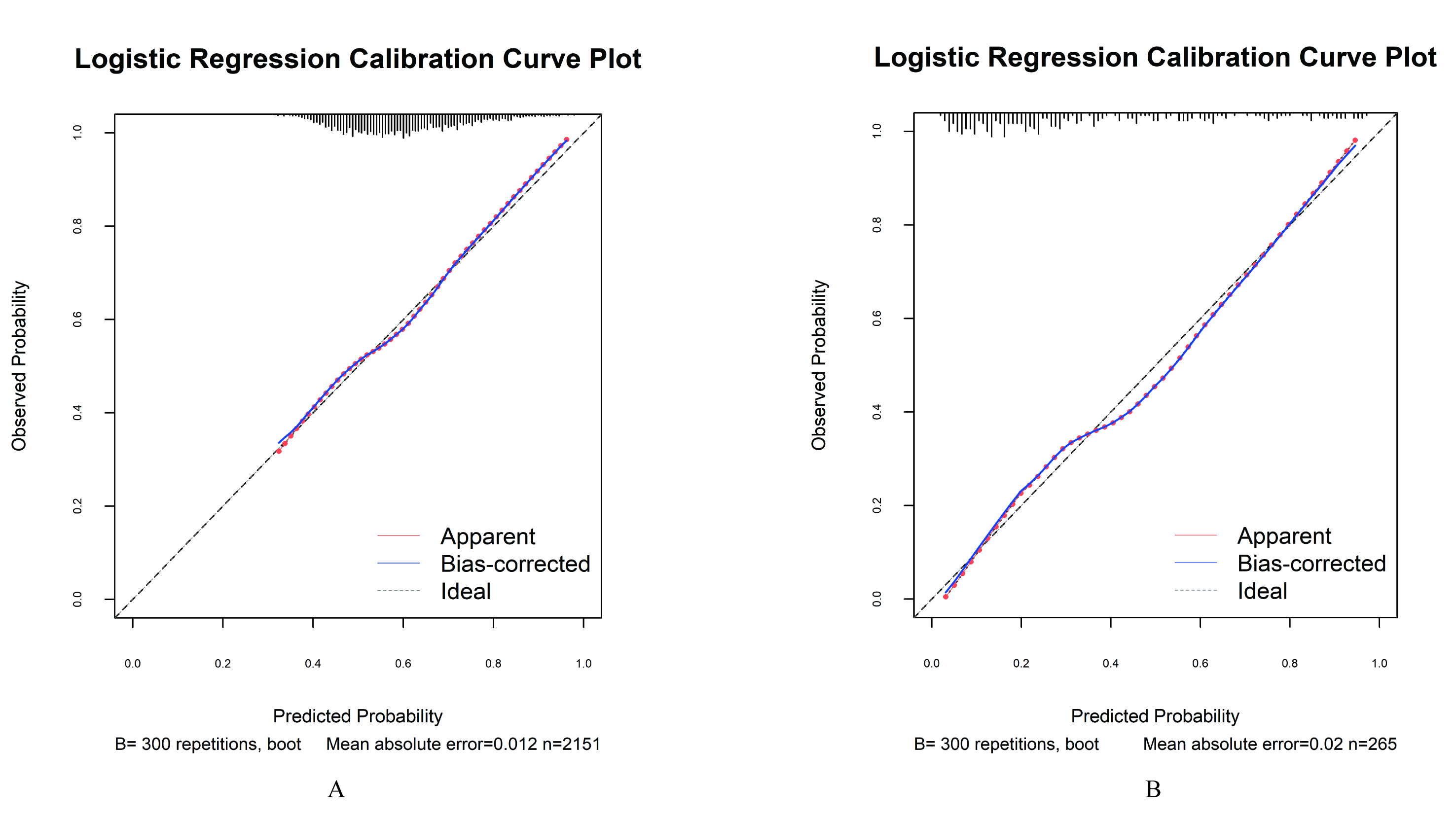 |
| --- |

Calibration plots (A) in MIMIC IV database (B) in the independent cohort.

**8.Supplementary Figure 2.**

| 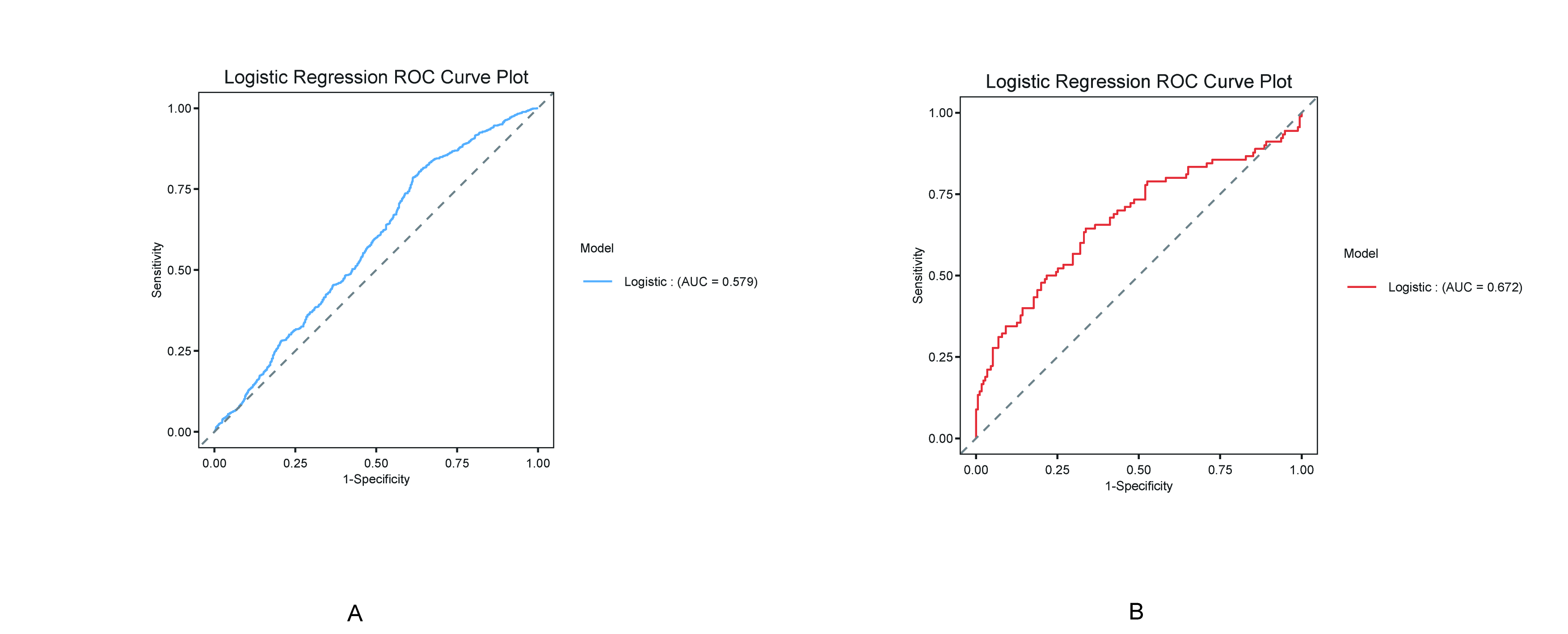 |
| --- |

ROC curve of GV for prediction of AKI (A) in MIMIC IV database (B) in the independent cohort.

**9.Supplementary Figure 3.**

| 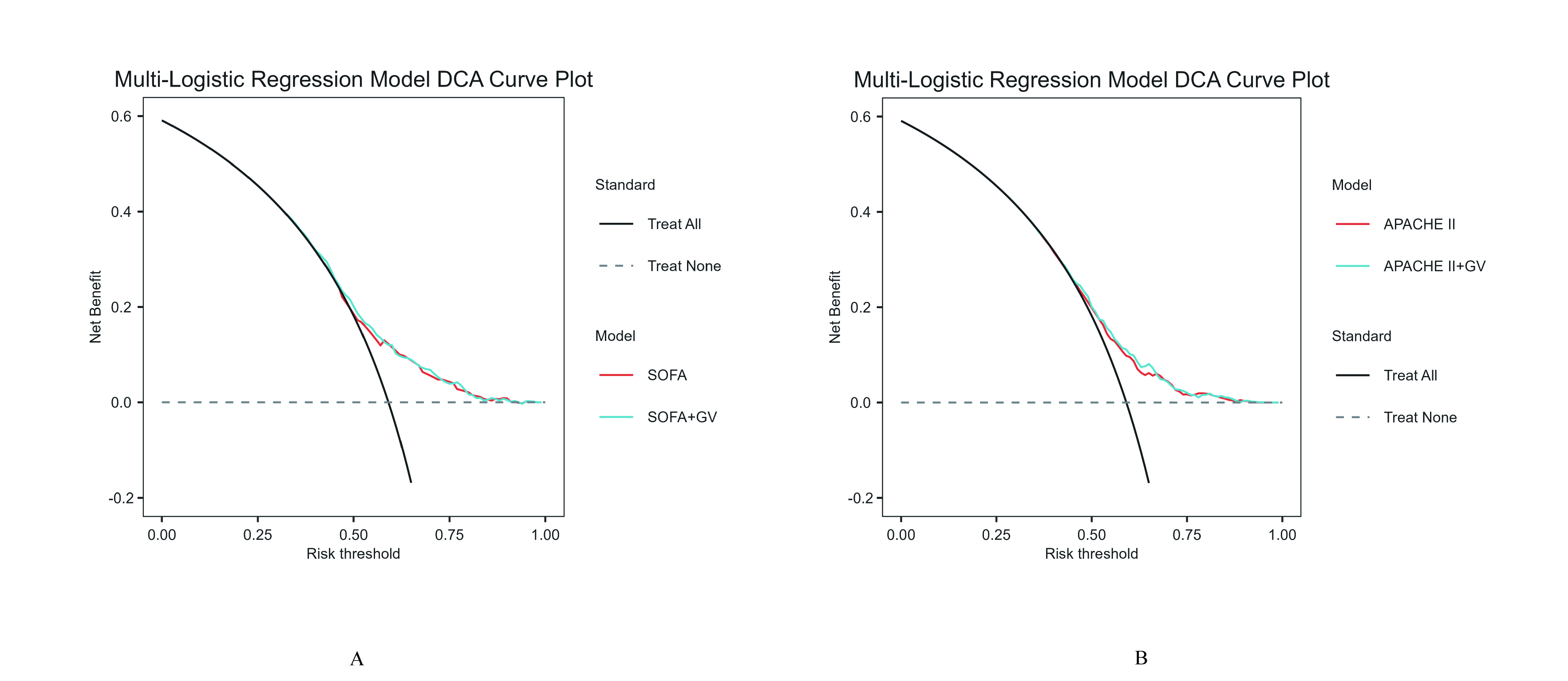 |
| --- |

Decision curve analysis of scoring tools (A) SOFA; (B) APACHE II with and without considering the GV in MIMIC IV database.

**10.Supplementary Figure 4.**

| 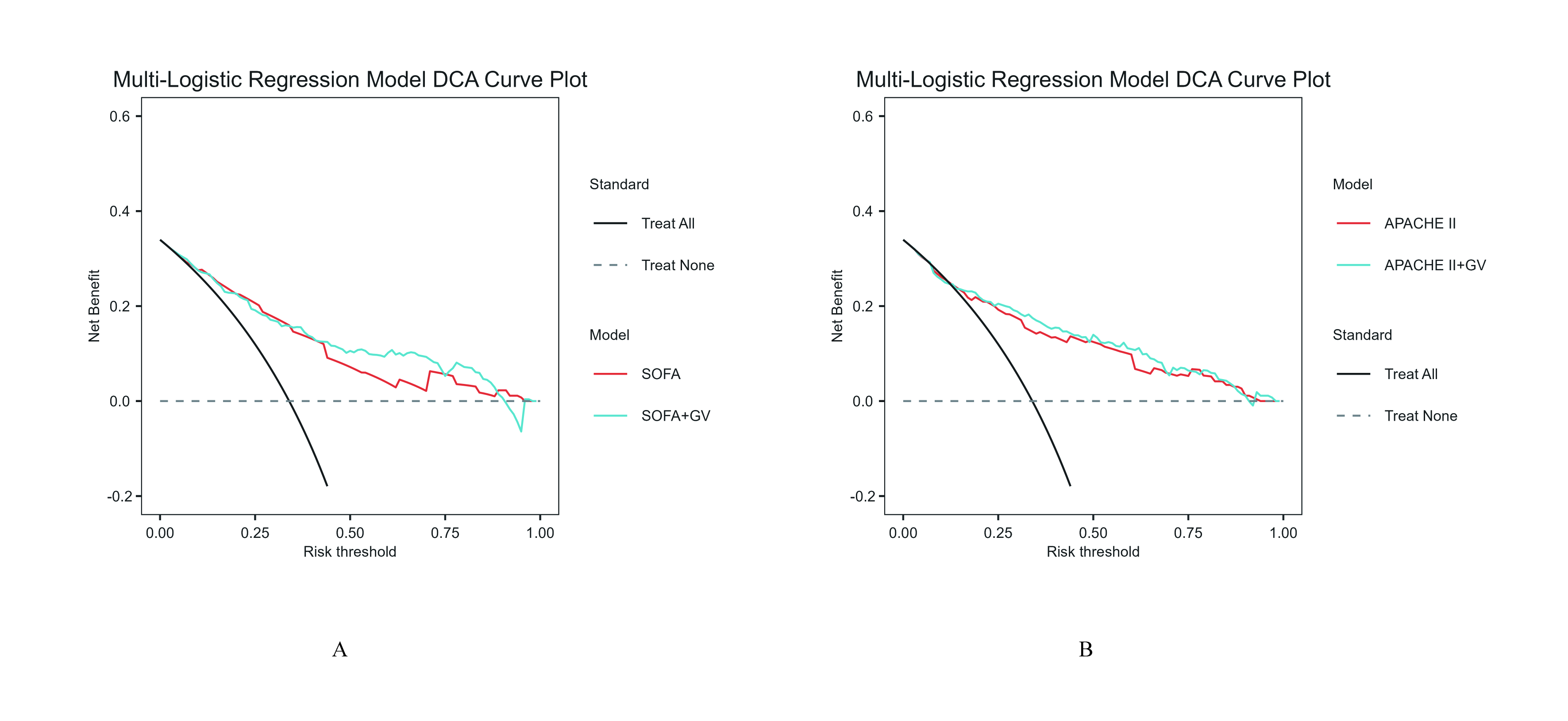 |
| --- |

Decision curve analysis of scoring tools (A) SOFA; (B) APACHE II with and without considering the GV in the independent cohort.

**11.Supplementary Figure 5.**

| 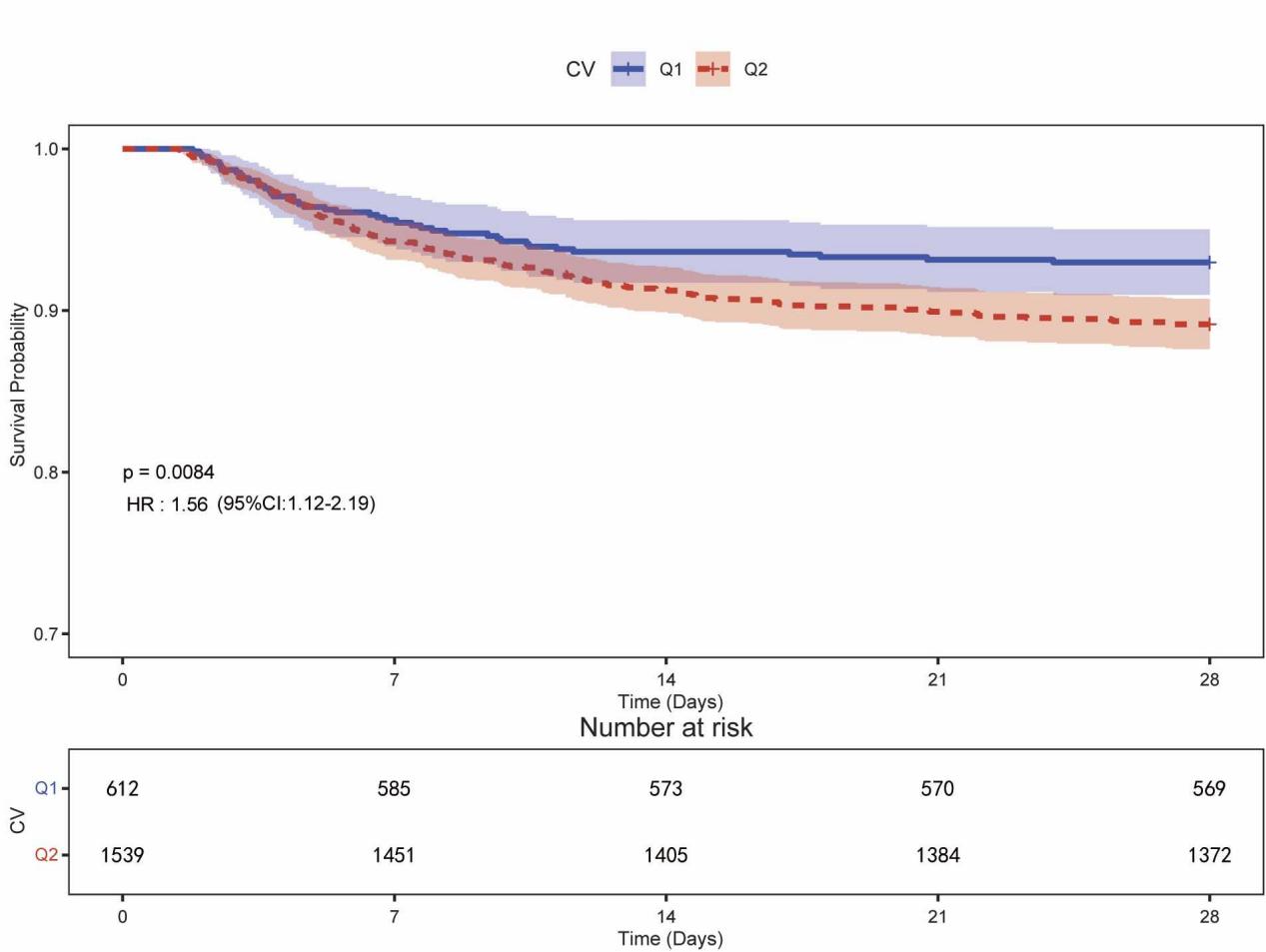 |
| --- |

Kaplan-Meier survival analysis curves for 28-day ICU survival stratified by CV: Q1 (<12.02), Q2 (≥12.02) in MIMIC IV database. The shaded areas represent the 95% confidence intervals.

**12.Supplementary Figure 6.**

| 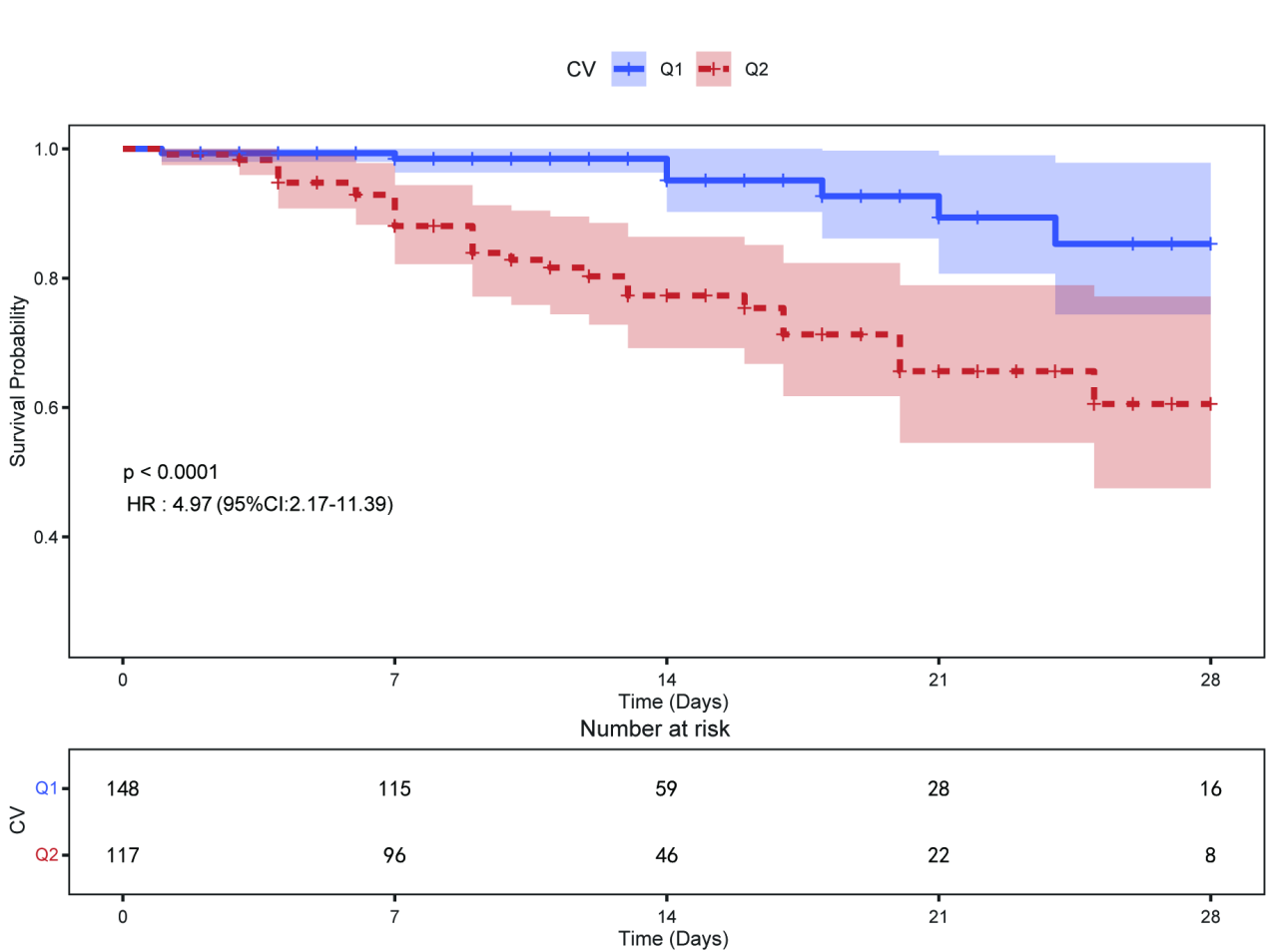 |
| --- |

Kaplan-Meier survival analysis curves for 28-day ICU survival stratified by CV: Q1 (<17.08), Q2 (≥17.08) in the independent cohort. The shaded areas represent the 95% confidence intervals.
