# Supplementary material for: Association between glycemic variability and the risk of acute kidney injury in patients with traumatic brain injury: a retrospective cohort study with independent cohort analysis
Source: Front Neurol. 2026 Apr 13;17:1797958. doi: 10.3389/fneur.2026.1797958 (PMC13114571; doi:10.3389/fneur.2026.1797958)
Supplement: Supplementary file 2 [file Table_1.docx]

**Abbreviations**

**traumatic brain injury TBI**

**acute kidney injury AKI**

**glycemic variability GV**

**Medical Information Mart for Intensive Care IV MIMIC-IV**

**coefficient of variation CV**

**receiver operating characteristic ROC**

**restricted cubic spline RCS**

area under the curve AUC

Kidney Disease Improving Global Outcomes KDIGO

interquartile range IQR

Multiple Imputation by Chained Equations MICE

continuous renal replacement therapy CRRT

Glasgow Coma Scale GCS

white blood cell count WBC

prothrombin time PT

activated partial thromboplastin time APTT

serum creatinine Scr

blood urea nitrogen BUN

hemoglobin HB

red blood cell count RBC

platelet count PLT

chronic heart failure CHF

chronic obstructive pulmonary disease COPD

reactive oxygen species ROS

systolic blood pressure SBP

mean blood pressure MBP

odds ratio OR

confidence interval CI

decision curve analysis DCA

Integrated Discrimination Improvement IDI

Net Reclassification Improvement NRI

mean amplitude of glycemic excursions MAGE

time in range TIR

standard deviation SD
